# The use patterns of novel psychedelics: experiential fingerprints of substituted phenethylamines, tryptamines and lysergamides

**DOI:** 10.1007/s00213-022-06142-4

**Published:** 2022-04-30

**Authors:** P. Mallaroni, N. L. Mason, F. R. J. Vinckenbosch, J. G. Ramaekers

**Affiliations:** Department of Neuropsychology and Psychopharmacology, Faculty of Psychology and Neuroscience, Maastricht University, P.O. Box 616, 6200 MD Maastricht, the Netherlands

**Keywords:** Psychedelic, Tryptamine, Lysergamide, Phenylethylamine, Novel psychoactive substance, Hallucinogen, 2–4-Bromo-2,5-dimethoxyphenyl)ethanamine (2C-B), 4-Acetoxy-N,N-dimethyltryptamine (4-AcO-DMT), 1-propionyl-lysergic acid diethylamide (1P-LSD)

## Abstract

**Background:**

Novel psychedelics (NPs) are an expanding set of compounds, presenting new challenges for drug policy and opportunities for clinical research. Unlike their classical derivatives, little is known regarding their use profiles or their subjective effects.

**Aims:**

The purpose of this study was to compile usage patterns and adverse event rates for individual NPs belonging to each of three main psychedelic structural families. Targeting the most widely used representatives for each class, we expanded on their phenomenological distinctions.

**Methods:**

A two-part survey was employed. We investigated the prevalence of novel phenethylamines, tryptamine and lysergamides in NP users (*N* = 1180), contrasting the type and incidence of adverse events (AEs) using a set of logistic regressions. Honing in on 2–4-Bromo-2,5-dimethoxyphenyl)ethanamine (2C-B) (48.6%), 1-propionyl-lysergic acid diethylamide (1P-LSD) (34.2%) and 4-Acetoxy-N,N-dimethyltryptamine (4-AcO-DMT) (23.1%), we examined their phenomenological separability using a gradient boosting (XGBoost) supervised classifier.

**Results:**

Novel phenethylamines had the highest prevalence of use (61.5%) seconded by tryptamines (43.8%) and lysergamides (42.9%). Usage patterns were identified for 32 different compounds, demonstrating variable dosages, durations and a common oral route of administration. Compared to phenethylamines, the odds for tryptamines and lysergamides users were significantly less for overall physical AEs. No significant differences in overall psychological AEs were found. Overall model area under the curve (AUC) stood at 0.79 with sensitivity (50.0%) and specificity (60.0%) for 2C-B ranking lowest.

**Conclusion:**

NP classes may hold distinct AE rates and phenomenology, the latter potentially clouded by the subjective nature of these experiences. Further targeted research is warranted.

**Supplementary Information:**

The online version contains supplementary material available at 10.1007/s00213-022-06142-4.

## Introduction

The subjective qualities of a drug often mould its notoriety. Such attributes are notably associated with the psychedelic class of psychoactive substances. Described as ‘mind-manifesting’ substances, psychedelics are characterised by profound distortions in sensory perception and subjective experience of one’s self, as well as alterations in mood, cognition and thought (Nichols 2016; Preller and Vollenweider 2016). A growing body of evidence has indicated that classic psychedelics such as psilocybin, dimethyltryptamine (DMT), lysergic acid diethylamide (LSD) and mescaline are safe and may be of clinical use for a range of psychiatric disorders (Chi and Gold 2020), bringing forth significant clinical and public interest, with their rising use (Yockey et al. 2020; Yockey and King 2021) hotly discussed by a diaspora of media (Aday et al. 2019). That said, classic psychedelics are currently scheduled as drugs of abuse under most national drug policies (Belouin and Henningfield 2018) and are thus illegal to purchase or manufacture. This discrepancy between growing public interest and lack of availability may arguably be fuelling a marketplace for accessible psychedelic counterparts, which circumvent existing legislation.

Novel psychedelics (NPs) are defined by regulatory authorities as novel psychoactive substances: drugs not controlled by the 1961 United Nations Single Convention on Narcotic Drugs or the 1971 United Nations Convention on Psychotropic Substances, yet are liable to abuse and/or dependence, producing similar effects to scheduled compounds (Schifano et al. 2015). NPs are typically synthetic pharmacophores of classical psychedelics, with their entry into the recreational market traced back to the pharmacopea of compounds published by Alexander Shulgin, detailing the effects and synthesis routes of over 200 novel hallucinogens, deliriants and stimulants (Shulgin and Shulgin 1991, 1997). Consequently, NPs are often more accessible than their Schedule I counterparts, sold online under a number of aliases (Schmidt et al. 2011) (Schmidt et al. 2011; Smith et al. 2015; Miliano et al. 2018) and gaining traction among recreational users (Neicun et al. 2020). However, they are distinct from their progenitors in that they lack a long history of human use and substantial research data. While prior work has begun to apply risk-classifications on the basis of individual clinical reports (Bersani et al. 2014; Corkery et al. 2020; Nugteren–van Lonkhuyzen 2020), and investigated both the possibility of enhanced likelihood of reduced mental wellbeing or potential therapeutic benefits pertaining to their use, a fine-grain understanding of individual NP use patterns has yet to be compiled. Quantitative descriptions of dose, route of administration, duration of effects and experience of (sub)acute psychological and physiological risks are the first key steps in informing harm reduction approaches.

Currently legislative strategies regarding NP regulation consist of a ‘cat and mouse chase’ in which attempts to restrict the use of a particular NP are met with the appearance of several-fold more, spreading toxicological evaluations thin. In this regard, consideration and identification of the family of chemical compounds that an NP falls in may be informative. Namely, novel (and classical) psychedelics are typically segregated into three structural families: tryptamines such as 4-AcO-DMT, lysergamides such as LSD and the NP 1P-LSD or phenethylamines such as mescaline and 2C-B (Nichols 2012). While the primary mechanism of action for their hallucinogenic effects in humans is attributed to serotonin 5-HT_2A_ agonism (Kometer et al. 2013; Preller et al. 2018; Nutt et al. 2020), accumulating evidence also emphasises the role of differential binding profile action at secondary sites such as serotonin 5HT_2C_ and 5HT_1A_ receptors, dopaminergic receptors and involvement of the glutamatergic system (Ray 2010; Studerus et al. 2012; Mason et al. 2020; Vollenweider and Preller 2020). As the families are differentiated via structure, they are proposed to have different binding affinities at both primary (5-HT_2A_) and secondary sites, resulting in differing in levels of potency, effect duration and likely subjective effect profiles (Leth-Petersen et al. 2014; Halberstadt et al. 2020), the latter of which is closely tied to outcomes in classical counterparts. Namely, experiential facets such loss of oneself and sentiments of unity and harmony have repeatedly been shown to drive positive psychological markers in studies employing healthy and clinical populaces (Roseman et al. 2018; Yaden and Griffiths 2020). Concerning this aspect, data-driven approaches using machine learning (ML) have proven themselves to be particularly sensitive in demonstrating structural-experiential alignment of psychedelics regarding the semantic content of these experiences (Zamberlan et al. 2018; Martial et al. 2019). By operating in an agnostic manner to capture non-linear multi-dimensional interactions and infer the degree of class ownership, these tools may be better suited to explain the distinctions between structural classes than hard, binary decision-boundaries set by a-priori assumptions in classical hypothesis-testing approaches (Rutledge et al. 2019; Li and Tong 2020). Decision-tree ML models are particularly favourable when seeking to explain variables of interests from non-normally distributed data such as self-reported independent subjective experiences and derive good explanatory value even in the presence of major scoring noise (Shanthini et al. 2019). Together with their redeployable nature once trained, they be may useful tools to generalise measures of subjective effects.

Thus, the question arises if the different psychedelic families also have different risk and benefit profiles. Quantitatively comparing the propensity of adverse side effects, as well as the subjective effect profile of the different families of NPs, may elucidate important factors to consider regarding identifying concerns of emerging NPs. Paired with information regarding current use practices, such findings would serve as a first step to focus future studies onto specific NP families, ultimately helping derive which classes may be most relevant for clinical study. The aims of the present survey study were therefore twofold. First, we aimed to establish current patterns of NP use as to assess whether the propensity for adverse effects differentiated between NP classes. Second, taking into consideration the importance of the experiential aspect of psychedelics, we explored the phenomenological separability of each class using a set of representatives for tryptamines (4-AcO-DMT), lysergamides (1P-LSD) and phenethylamines (2C-B). By using an extreme gradient boosting XGBoost algorithm, we highlight the suitability of exploratory ML approaches for the study of subjective drug effects.

## Methods

### Design

The study employed an unincentivised, anonymous online survey, promoted as an investigation into the use and effects of novel psychedelic substances. Advertisements were placed on Internet fora related to psychedelic drug use, such as psychonautwiki.org and Open Foundation. The survey was regularly disseminated on discussion boards pertaining to NPS use including Bluelight.com, Reddit (R/ResearchChems, R/Psychedelics/ etc.) and Drugsforum.nl. The eligibility criteria for participation consisted of being 18 years or older, having experience with a novel psychedelic substance and providing informed consent. Ethical approval was obtained from the Ethics Review Committee of Psychology and Neuroscience of Maastricht University (ERCPN- 222_77_04_2020).

The survey was created and hosted on the Qualtrics software platform (XM 12). As to gather information on general use frequencies and information pertaining to qualitative components of NPS effects, the survey was subdivided into two sections: a first half pertaining to general use and a second revolving around a recent (< 6 months) full-dose experience with a novel psychedelic.

Between May 2020 and January 2021, 2700 responses were collected of which 1180 respondents were 18 years or older, provided informed consent and completed the first half of the questionnaire. Of these, a subset of 599 respondents provided information pertaining to a recent experience with an NPS. The duration of the survey was dependent on the number of drugs a respondent chose to provide information on as well as their choice to continue with questions pertaining to a recent experience. It was possible to pause the survey and complete it at another time. The average survey completion time was 15 minutes.

### Measures

#### Demographics

Background information collected consisted of age, biological sex, highest education level achieved and continent of residence. Classical psychedelic use history was assessed by providing a selection of substance choices: psilocybin, MDMA, ayahuasca, DMT, LSD, mescaline and the alternative option of no prior experience.

#### General NPS use

Participants were first asked about their previous experience with each of the three structural families of psychedelics, followed by the option to provide information particular to a listed example. Each choice was precluded with examples of representatives for each class. Drug selection was centred around previously documented, recreationally used novel psychedelics of which 16 phenethylamines, 13 tryptamines and 4 lysergamides were selected (for a complete list all compounds, see Table 1). Choices for each structural family were supplemented by an ‘other’ text option to provide the opportunity to include an unlisted substance.

**Table 1:** Listed survey NPS choices. Each choice made available to users to select from are organised according to their structural sub-specifications.†While 5-MeO-DMT is a natural indolealkylamine extracted from *Bufo alvarius* toad venom (Weil and Davis 1994), prior investigations have classified it as an NPS (Khaled et al. 2016)

Phenethylamines	Tryptamines	Lysergamides
*N-(2-methoxybenzyl) phenethylamines (25X-NBOMes)*	*N, N-diisopropyltryptamines*	*LSD analogues*
25b-NBOMe	4-HO-DiPT	1P-LSD
25c-NBOMe	4-AcO-DiPT	ALD-52
25i-NBOMe	DiPT	AL-LAD
	DPT	LSA
*Substituted dimethoxyphenethylamines*		
*(2C-Xs)*	*N-methyl-N-isopropyltryptamines*	
2C-B	MiPT	
2C-C	5-MeO-MiPT	
2C-D	4-HO-MiPT	
2C-E		
2C-I	*N, N-diallyltryptamines*	
2C-P	5-MeO-DALT	
2C-T-2		
TCB2	*Psilocin derivatives and homologues*	
Bromo-DragonFly	4-AcO-DMT	
	4-AcO-MET	
*4-Substituted-2,5-dimethoxyamphetamines (DOx)*	4-HO-DET	
DOB	4-HO-MET	
DOC		
DOI	*N, N-dimethyltryptamine derivatives*	
DOM	5-MeO-DMT†	

Binary (yes/no) questions were employed to evaluate the occurrence of clinically relevant psychological and/or physical adverse events (AEs), each of which was supplemented by additional subcategories (Physical: Gastrointestinal, Cardiovascular, Seizures; Psychological: Anxiety, Paranoia, Low mood). The choice of these subtypes was defined by prior literature on serotonergic classical and novel psychedelics (Nichols 2016; Dos Santos et al. 2018; Luethi and Liechti 2020). As a follow-up, we asked users whether these effects overall occurred acutely or long term (after the dissipation of drug effects).

### Recent NP experience

Upon completion of the first half of the survey, respondents were offered the possibility of providing information on a particular psychedelic experience they had in the last 6 months using an NP. It was stressed that their choice should consist of a “full” experience (one of noticeable perceptual effects). Their choice was facilitated by providing a list of all previously suggested NP representatives, alongside an “other” category. Due to the expected popularity of 2C-B and 4-AcO-DMT and with the large number of choices made available in the survey, an option was provided to encouraging users to detail one or the other. Following their selection of a compound, respondents were once again prompted for the estimated dose of this full experience.

As to identify the experiential components that define the phenomenology of a particular NP experience, participants were subject to standardised questionnaires assessing drug effects retrospectively. Employed in clinical trials evaluating the acute effects of psychoactive drugs, these also serve the dual purpose of establishing qualitative reference points for data on yet-trialled NPS. The 5D-ASC scale measures altered states of consciousness and contains 94 items in the form of visual analogue scales. The instrument consists of five dimensions comprising Oceanic Boundlessness, Anxious Ego Dissolution, Visionary Restructuralisation, Vigilance Reducton, Auditory Alterations and 11 subscales (Studerus et al. 2010). The nature of these subscales is described in the supplementary materials. The 5D-ASC has been validated using a range of hallucinogens, entactogens, stimulants and non-pharmacological altered states of consciousness (Liechti et al. 2017; Mueller et al. 2018; Kuypers et al. 2019; Luke et al. 2019; Holze et al. 2020; Mason et al. 2020; Uthaug et al. 2021).

Efforts are underway to produce a compendium of drug phenomenology (Schmidt and Berkemeyer 2018) as to establish points of reference. To extend the generalisability of potential findings, respondents were also asked to complete in supplement the 48-item Addiction Research Centre Inventory (ARCI) (Martin et al. 1971), previously employed in studies of NPs and other psychoactives (Papaseit et al. 2018; Papaseit et al. 2020). Going further, as subjective experiences under psychedelics are coloured by extraneous contextual factors such as set and setting (Hartogsohn 2016). We provide in addition as control variables, use motivation as assessed in prior evaluations of NPs endorsed motives (Kettner et al. 2019) alongside details regarding the environment in which this recent experience took place. Methods and results pertaining to these inventories can be found in the supplementary materials.

### Statistics

#### General

Survey data were cleaned using SPSS Version 24.0. Respondents who failed to complete the first half of the survey were excluded.

Follow-up questions on a chosen substance were retained based on dose validity. As mean recreational doses are likely subject to significant variance due to intraindividual motives, tolerance and lack of exact dose knowledge, we characterised outliers as incorrectly used mass (mg/g/μg) metrics. Shapiro-Wilks tests were conducted prior to analyses to examine the homogeneity of variance for all continuous variables. Incorrectly used metric outliers were defined by (1) visual identification (ex: 1000 g) and (2) a Box-Cox power transformation followed by *Z*-score rescaling. Points found to be ≥ than 3 S.D were excluded. Proportions (%) are reported for sex, gender, education, continent of origin, education-level and classical psychedelic history. Mean (± *SD*) is given for age. Frequencies and proportions presented for individual drug use results are weighed according to the total respective family sample size (phenethylamines, tryptamines, lysergamides). In the case of follow-up questions, proportions are listed in relation to the individual drug sub-population. In the case of dose, results are reported as the median dose alongside the interquartile range (IQR). For all tests, statistical significance was defined by* p* < 0.05.

#### Class and drug comparisons

A set of logistic regression analyses were employed to investigate associations of our dependent variables of interest: (1) incidence of physical side effects; (2) incidence of psychological side effects; (3) type of adverse effect (Physical: gastrointestinal, cardiovascular, seizures; Psychological: anxiety, paranoia, low mood); and (4) the duration of the side effect (acute/long-term).

Due to the nature of the study, no suitable drug-naïve reference group was available. As such, in all regression models, phenethylamines were set as the intercept (*β*_*0*_), having the largest number of observations to ensure sufficient statistical power. For each model, we included age and biological sex as confounders. Consequently, adjusted odds ratios (aORs) are reported therein. Being aware that the orthogonality of our predictors may be affected by the nested, multi-choice format of our survey, we calculated each of their variance inflation factors (VIF) (Midi et al. 2010). When contrasted to phenethylamine use, neither tryptamine use (1.24) nor lysergamide use (1.23) produced scores beyond a conservative 2.5 VIF threshold, reflecting low collinearity.

#### Supervised classification of subjective drug effects

The classification algorithm was trained and validated using the most popular compound of each structural family, defined by the largest number of observations. Our choice of canonical class members was narrowed down to 2C-B (phenethylamines, *n* = 176), 4-AcO-DMT (tryptamines, *n* = 59) and 1P-LSD (lysergamides, *n* = 102). Due to their diminished dimensionality, we trained our model on the five core facets of the 5D-ASC. Model development and evaluation were conducted using the following Python toolkits: Imbalanced-learn and Scikit-learn (Pedregosa et al. 2011). We selected for Extreme Gradient Boosting (XGboost), a decision tree-based machine learning method (Chen and Guestrin 2016) to build the classifier algorithm. This was based on its robustness to feature multicollinearity, inherent feature selection, capacity to handle sparse data and detect non-linear relationships between variables. Furthermore, XGBoost’s inherent design allows for high interpretability: by employing a recursive tree-based decision system in which several weak trees are combined in order to generate a collectively strong model, the importance of each individual feature used is determined by its accumulated use in each decision step in trees. This computes a metric characterising the relative importance of each feature for each learning step, otherwise absent in other ML approaches. This feature importance is valuable for estimating features that are the most discriminative of model outcomes, especially when they are related to meaningful clinical parameters.

Datasets with low numbers of observations and numerous dependable variables are often subject to overfitting (Ying 2019). We therefore took pre-processing steps to normalise and resample features as to improve model generalisability prior to training (these details can be found in the supplementary materials). Controlling model bias-variance trade-off is a key task in machine learning (Cawley and Talbot 2010). One optimal approach to this is nested cross-validation (CV), an equivalent to creating multiple train-test splits to derive robust estimates of model predictive performance in unseen data (Varma and Simon 2006; Krstajic et al. 2014). Following pre-processing, we used a tenfold nested *K*-fold CV (Scikit-learn), wherein at each iteration, 5 of the folds were used in the inner loop to tune model parameters and train the algorithm, and the 5th fold was used in the outer loop to test the trained model. Tuned model parameters included the number of trees (100 to 1000), tree depth (1, 2 or 3 to allow for higher order interactions) and the learning rate (0.1 to 0.3). Training of the XGBoost model was based on this tenfold stratified CV repeated 3 times, using the average AUC of all possible pairwise combinations of classes (Hand and Till 2001). The area under the receiver operating characteristic curve (AUROC), or AUC, was calculated for each class. AURC provides an aggregate measure of performance across all possible classification thresholds, by contextualising sensitivity (sensitivity) as a function of the non-specificity (1 — specificity) for a classifier as classification thresholds are varied. To aid in interpretation, Cohen’s *d* equivalents of AUC scores list an AUC = 0.58 as a small effect size (0.2), AUC = 0.69 a medium effect size (0.5) and AUC = 0.79 a large effect size (0.8) (Salgado 2018). For completeness, we report model feature importance, class-specific and macro-average F1 scores, precision and recall. Definitions for each additional measure and a description of the model are found in the supplementary materials.

While ML multivariate approaches offer the opportunity to derive latent patterns on yet-seen data, they perform blind to the underlying distribution, working on approximations derived from training data. As such, ML models may form assumptions about a population which may not be representative of the true sample distribution (Li and Tong 2020). To cross-examine whether flagged model distinctions stemmed from random phenomena, we performed nonparametric, univariate Kruskal–Wallis one-way analysis of variance tests to confirm group differences. For completeness, post hoc multiple comparisons were performed using Bonferroni-corrected (*p* < 0.017*)* pairwise Dunn’s tests, described in the supplementary materials.

## Results

The final sample of 1180 respondents consisted of 994 males (84.2%) and 186 females (15.8%) with a mean age of 26.4 (SD: 8.4 range 18–64). Most of the sample had reached a tertiary level education at a university/trade school/college (68.4%), seconded by a high school diploma/equivalent (29.9%) and followed by primary/elementary education (1.7%). Participants were based in North America (50.7%), Europe (45.3%) and Oceania (2.5%) with a minority from South America (0.8%), Asia (0.5%) and Africa (0.2%). The vast majority of respondents (96.8%) had previously used a classical psychedelic, including psilocybin (80.8%), MDMA (76.9), DMT (40.7%), mescaline (19.10%) and ayahuasca (8.3%). Respondents were poly-users, with 85.7% of the sample having tried more than one classical psychedelic, an average of 3.1 (SD: 1.4).

### General NPS use

#### Frequency of use

**Fig. 1:**
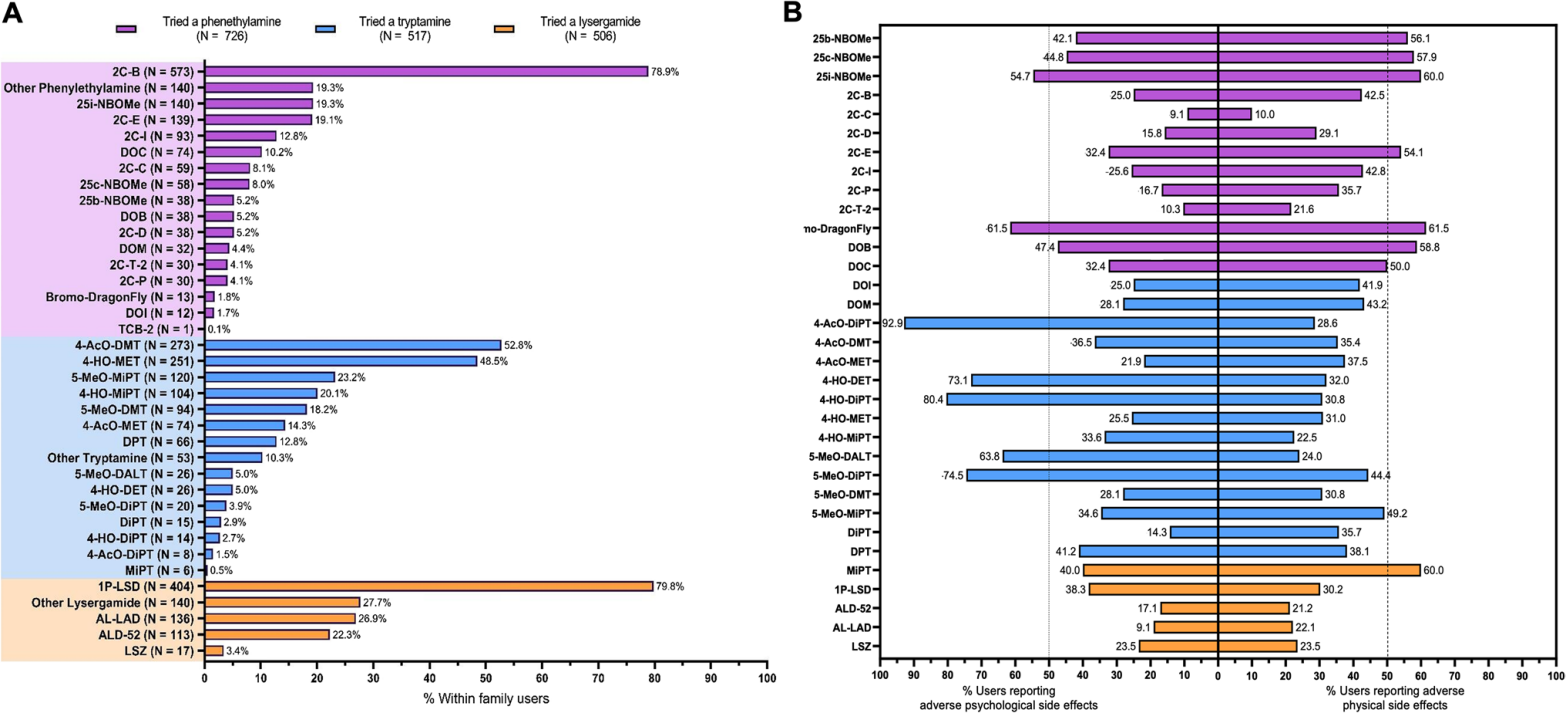
Self-reported NPS use and adverse effects per structural family. **a** Percentage of NPs reported to have been previously tried by respondents. In (**b**) can be seen the incidence rate of adverse physical and psychological side effect for each drug. For both (**a**) and (**b**), proportions are listed in relation to each colour-matched family sample size

Of the three main families of NPS, phenethylamines had the highest prevalence of use (61.5%), seconded by tryptamines (43.8%) and lysergamides (42.9%). While a variety of drugs were reported to have been tried, 2C-B was the most used NP (48.6%), followed by 1P-LSD (34.2%) and 4-AcO-DMT (23.1%). Raw percentages and frequencies for prior NPS use can be found in Fig. 1a. Users had experience with a range of novel psychedelic drugs, trying an average of 5.9 (SD: 4.0) of the 33 available compounds.

For each of the classes, several respondents chose to provide an alternative substance, accounting for 19.3%, 10.3% and 27.7% of phenethylamines, tryptamines and lysergamides respectively. Recurring compounds included the phenethylamines 2C-B-FLY (26.4%) and 25e-NBOH (6.4%), the tryptamines MET (13.2%) and 4-AcO-DET (11.3%), and the lysergamides 1cP-LSD (45.7%) and ETH-LAD (34.2%). Written-in responses were excluded from the ensuing report due to their large heterogeneity in the number of compounds listed at one time. TCB-2 was not included in subsequent reporting due to a lack of observations (0.1%).

#### Patterns of use

**Fig. 2:**
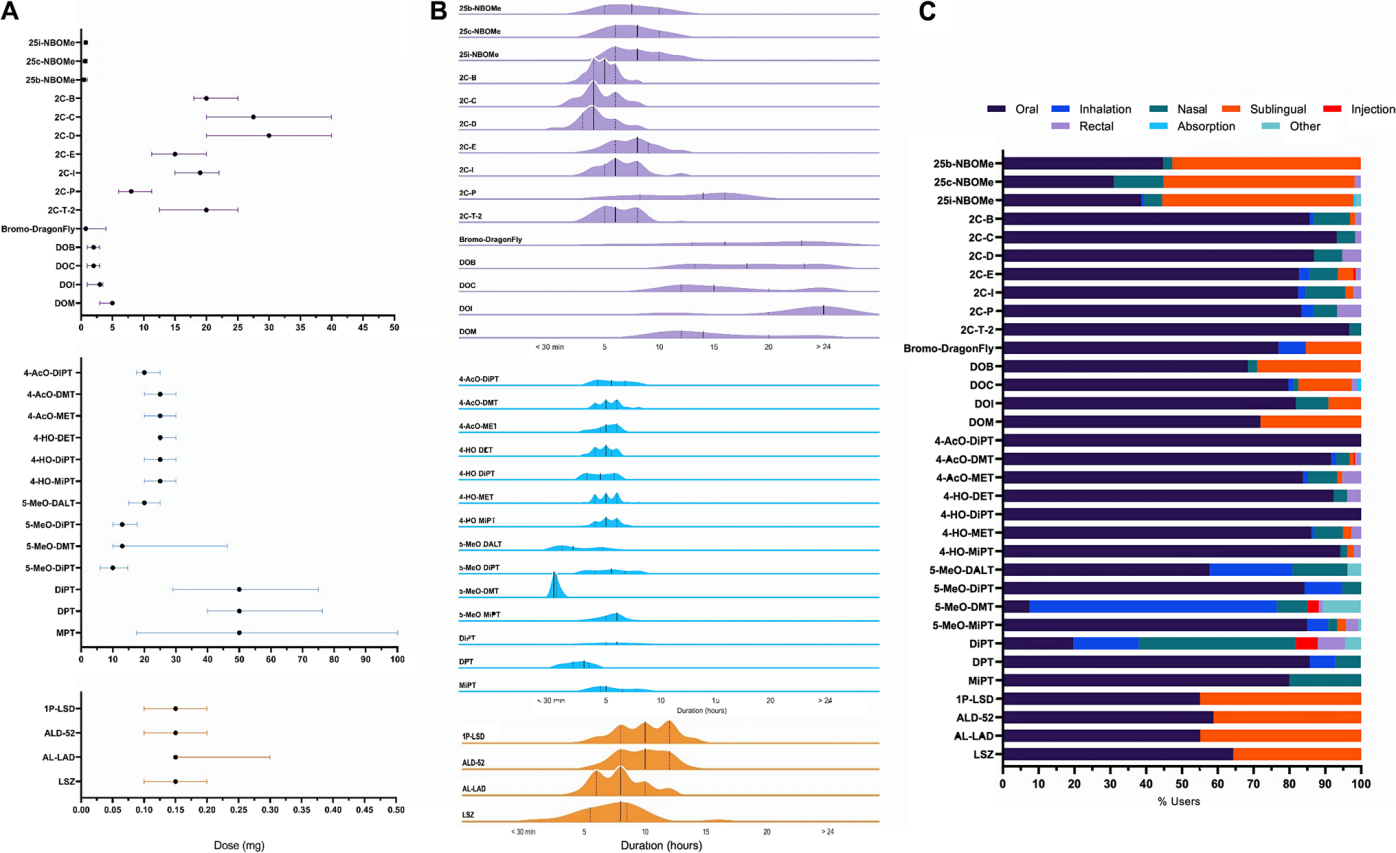
Patterns of NPS use relating to dose, duration and mode of administration. **a** depicts median dosage (mg) and the IQR for each drug across all modes of administration, and (**b**) shows a ridge plot of mean drug effect duration across all modes of administration. Filled lines represent the median, and dotted lines reflect the IQR. A smoothing kernel of 0.7 was applied for this visualisation. (**c**) Administration routes in proportion of individual NP use

Users reported a large range of doses for each compound. Due to the skewed nature of the data, median doses (mg) and their interquartile range (IQR) across all modes of administration are reported in Fig. 2a.

The substituted phenethylamine class of NBOMe’s such as 25i-NBOMe (median: 750.0 μg, IQR: 400.0) and lysergamides such as 1P-LSD (median: 150.0 μg, IQR: 100) had the highest overall self-reported potency, as indicated by the notable microgram range of doses. Conversely, for each of their respective classes, the 2C-X compounds such as 2C-D and the lysergamide AL-LAD were the upper scales of identified doses. Most notably, tryptamines presented the highest recreational listed doses, with DPT users reporting the largest used dose (median 50 mg, IQR: 40). Data pertaining to the mean reported doses per route of administration are found in the Table S1.

Similarly, effect duration was stratified across drug families. For each drug, durations are represented as frequency density estimates in Fig. 2b alongside their IQR. Median effect duration for phenethylamines was found to be 6 h whereas for lysergamides, it was 10 h and tryptamines 4 h. These durations were further differentiated according to the nature of the drug, with users of the DO-X compound DOI reporting the longest lasting effects with a median effect duration of more than 24 h whereas the shortest being for the tryptamine 5-MEO-DMT at < 30 min. Novel psychedelics were reported to have been taken in a variety of ways. Oral intake was the most frequently reported mode of administration across all phenethylamines (69.7%), tryptamine (65.8%) and lysergamide users (56.7%). Noteworthy contenders included sublingual intake for phenethylamines (13.7%) and lysergamides (43.3%), whereas for tryptamines, inhalation comprised the second most popular option (15.3%). Where available, median doses and durations separated by each mode of administration are reported in the supplementary materials Tables S1 and S2.

#### Side effects

For each drug, users were asked if they had previously experienced any overall physical or psychological side effects (Fig. 1b).

##### Physical side effects

Binary logistic regression analyses (Table 2) revealed that in contrast to phenethylamines, lysergamides (aOR = 0.53; *p* < 0.001, 95% CI [0.43–0.66]) and tryptamines (aOR = 0.38; *p* < 0.001, 95% CI [0.31–0.47]) users reported significantly less overall physical AEs.

**Table 2:** Odds ratios for tryptamines and lysergamide adverse event incidence, type and duration in comparison to phenethylamines. Adjusted odds ratios (aORs) for each dependent variable are listed with confidence intervals ([CI] *p < 0.05, **p < 0.001, ***p < 0.001. *p* values for intercept significance are listed in the same manner. Cell counts < 30 are left blank (.) due to extreme heteroscedasticity

	Phenethylamines *β*_*0*_intercept *p*	Tryptamines aOR [CI]	Lysergamides aOR [CI]
Physical adverse effects	***	0.38 [0.31–0.47]***	0.53 [0.43–0.66]***
Gastrointestinal	***	0.48 [0.38–0.59]***	0.10 [0.78–1.26]
Cardiovascular	***	0.42 [0.32–0.56]***	1.23 [0.91–1.17]
Seizures	***	0.23 [0.08–0.91]*	0.045 [0.01–0.14]***
Acute	***	0.43 [0.35–0.53]***	1.144 [0.91–1.43]
Long term	·	·	·
Acute and long term	·	·	·
Psychological adverse effects		0.85 [0.69–1.045]	0.92[0.74–1.14]
Anxiety	***	0.88 [0.71–1.10]	1.49[ 1.18–1.88]***
Paranoia	***	0.96 [0.72–1.27]	1.62 [1.20–2.20]**
Low mood	***	0.63 [0.42–0.94]*	0.33 [0.22–0.48]***
Acute	**	0.809 [0.65–1.001]	1.128 [0.90–1.41]
Long term	**	·	0.88 [0.57–1.37]
Acute and long term	*	0.35 [ 0.16–0.75]**	0.793 [0.40–1.58]

At a compound level, for phenethylamines, physical AEs were most frequently reported by Bromo-Dragonfly users (61.5%), 25i-NBOMe (60%) and DOB (58.8%) users. As for lysergamides, these were most frequently reported by 1P-LSD (38.3%), LSZ (23.5%) and AL-LAD (19.1%) users, whereas MiPT (60%), 5-MeO-MiPT (49.2%) and 5-MeO-DiPT users (44.4%) represented the highest incidence rates for tryptamines. In comparison to phenethylamines, risk of gastrointestinal and cardiovascular side effects were significantly lower for tryptamines ((aOR = 0.48; *p* < 0.001, 95% CI [0.38–0.59]) and (aOR = 0.42 (*p* < 0.001, 95% CI [0.32–0.59])). Tryptamines were significantly less likely to produce seizure-type AE; aOR 0.23 (*p* < 0.05, 95% CI [0.08–0.59]; with lysergamides having the lowest reported odds of aOR = 0.04 (*p* < 0.001, 95% CI [0.01–0.14]). Overall, the acute incidence of physical side effects associated to tryptamine use was significantly less likely than that for phenethylamines (aOR = 0.43 (*p* < 0.001, 95% CI [0.32–0.59])) Due to extreme heteroscedasticity, maximum likelihood estimation for models pertaining to extended AE duration (long term/both) is not reported.

##### Psychological side effects

Odds ratios for phenethylamines, tryptamines and lysergamides did not significantly differ between groups in the case of overall psychological AEs (Table 2).

However, compounds expressed heterogeneous incidence rates. Once more, the phenethylamines Bromo-Dragonfly (61.5%), 25i-NBOMe (57.4%) and DOB (54.7%) had the highest rate of psychological AEs. Of all, the tryptamines 4-AcO-DiPT (92.9%), 4-HO-DiPT (80.4%) and 5-MeO-DiPT (74%) ranked highest. No individual lysergamide held an incidence rate over 50%, the highest scorers being 1P-LSD (38.3%), LSZ (23.5%) and AL-LAD (19.1%). Psychological effects did not significantly differ among classes (*p* > 0.1). However, the incidence of specific psychological AEs varied between compounds. Lysergamides were significantly more likely to produce anxiety (aOR = 1.49 (*p* < 0.001, 95% CI [1.18–1.88]) and paranoia (aOR = 1.62 (*p* < 0.01, 95% CI [1.20–2.20]) whereas both lysergamides (aOR = 0.33 (*p* < 0.001, 95% CI [0.22–0.48])) and tryptamines (aOR = 0.63 (*p* < 0.05, 95% CI [0.42–0.94])) showed significantly lesser odds of low mood than phenethylamines. Both acute and long-term psychological adverse events were significantly less likely for tryptamines in comparison to phenethylamines (aOR = 0.35 (*p* < 0.01, 95% CI [0.16–0.75])).

### Retrospective reports of novel psychedelic experiences

Of the total sample, 599 respondents (50.8%) chose to continue and provided details regarding a recent, full-dose, psychedelic experience with an NPS. Reflecting our previous general use findings, 2C-B, 1P-LSD and 4-AcO-DMT were the most frequently chosen options, at 29.4% (*N* = 176), 17% (*N* = 102) and 9.8% (*N* = 59) of the final sample respectively. The full scope of NPs listed by users can be found in Table S4.

#### Subjective effects of 2C-B, 4-AcO-DMT and 1P-LSD

**Fig. 3:**
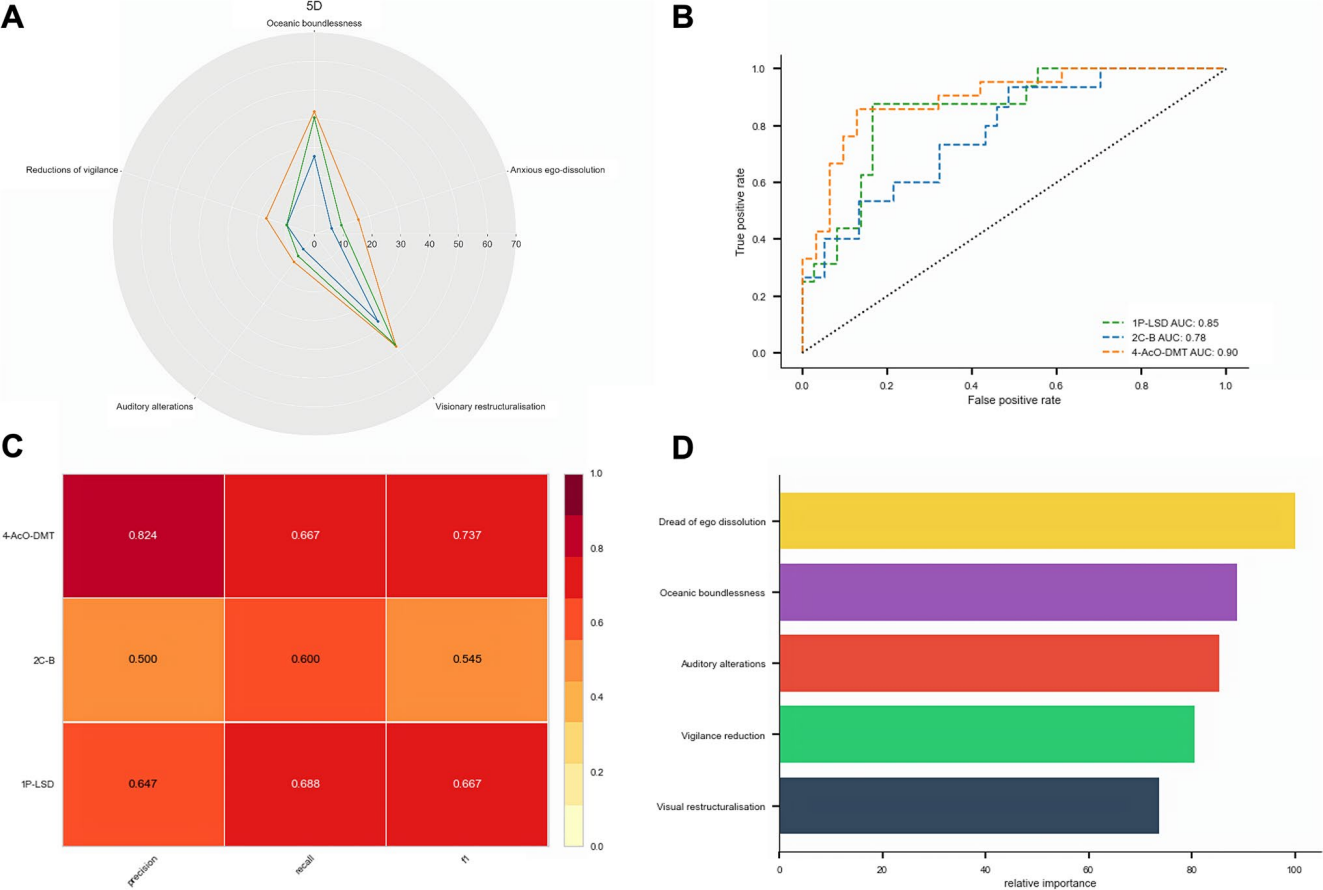
Subjective effect scores and XGBoost model performance. **a** Retrospective effects of 2C-B (*N* = 176), 4-AcO-DMT (*N* = 59) and 1P-LSD (*N* = 103) on the 5 major dimensions of 5D-ASC. Scores are calculated as percentage maximum scores. Data points show means; maximum score = 100. Scores pertaining to the 11 subdimensions of the 5D-ASC can be found in the supplementary materials. **b** ROC curves show the superiority in classifying each drug against all others. The corresponding values of AUC for each drug are presented, as well as their individual classification report in (**c**). In (d), the most important features of the XGBoost model are represented, as calculated by gain

The acute effects of a recent, full-dose experience with 2C-B, 4-AcO-DMT and 1P-LSD were retrospectively assessed using the 5D-ASC (Fig. 3a). Violin plots for all scales including the 11D-ASC and ARCI are provided in Fig. S4(a). Individual multiple regressions for each compound did not identify the presence of dose–response relationships for any of the scales (*p* > 0.1). Confirmatory Wilcoxon-signed rank testing (see supplementary materials) demonstrated significant main effects for each of the 5 main dimensions (*χ*^2^(2) = 10.9 *p* =  < 0.05–*χ*^2^(2) = 40.9 *p* =  < 0.001), reflecting non-spurious differences between groups.

#### Classification of subjective drug effects

The XGBoost algorithm was used to extract the optimal features needed to classify the three canonical novel psychedelics. The classification model considered each drug according to self-reported scores of the 5 dimensions of the 5D-ASC. Figure 3b presents the ROC curves; the corresponding values of area under the curve (AUC) for each drug, translating to an average macro-AUC of 0.79 [95% CI: 0.77–0.81] across all testing folds. Figure 3c represents the average main classification metrics, combined into a common F1-score per class.

Based on a probability threshold of 50%, the sensitivities (precision) were approximately 82%, 65% and 50% in the 4-AcO-DMT, 1P-LSD and 2C-B models, respectively. The specificities (recall) were approximately 67%, 69% and 54% in 4-AcO-DMT, 1P-LSD and 2C-B models, respectively. Macro-averages for all classes yielded an average model accuracy of 0.63 [95% CI: 0.61–0.64]. Sores for precision were 0.62 [95% CI: 0.61–0.64]; recall 0.62 [95% CI: 0.61–0.64] and for a combined f1 of 0.62 [95% CI: 0.60–0.63]. Together, these scores are symptoms of multiclass model performance being driven by above chance separability of 4-AcO-DMT and 1P-LSD across classification thresholds.

In this study, relative feature importance was calculated during the XGBoost model creation, as defined by the explained variance, each feature contributes to the decision tree branch it resides on. No feature was deemed irrelevant. Figure 3d shows the result of deriving the importance of the main features among all the explanatory variables. As shown, the most important features were *dread of ego dissolution and oceanic boundlessness. Visual* *restructualisation* contributed the least to the model.

## Discussion

The present study aimed to provide information surrounding individual NPs among 1180 adult users. By employing a two-part structure, we were firstly able to collect information regarding the prevalence, use profile and side effects of individual novel psychedelics, which allowed us to further home in on a popular triad of canonical NPs: 2C-B, 4-AcO-DMT and 1P-LSD. Considering their popularity, we collected comprehensive information on their experiential elements. Each a representative of the three main structural classes of psychedelics, we tapped into the detailed nature of the 5D-ASC questionnaire using a supervised classification technique to explore the distinguishability of their self-rated perceptual profiles.

Our sample comprised of mostly male, well-educated respondents who were previously acquainted with classical psychedelics and a range of NPs. Users most frequently stated prior experience with phenylethylamine derivatives (61.5%) with 2C-B representing the most frequently tried drug of all (48.6%). Despite only reflecting a snapshot of current NP use, our findings are consistent with prior epidemiological NPS surveys and EMCDDA novel psychoactive substance seizures by law enforcement (Neicun et al. 2020). That said, users also reported consuming an average of five different compounds. Written-in responses exemplify the continual evolution of NPs; with dihydrodifuran analogue 2C-B-FLY, accounting for 26.4% of all phenethylamine write-ins, synthesised not-long after the publication of Shulgin’s pharmacopeia (Monte et al. 1997) and 1cP-LSD (45.7% of unlisted lysergamides) being detected as recently as 2019 (Brandt et al. 2020). As governments signal their intention to control a substance, wholesale producers operating in legal grey areas can easily switch to new, noncontrolled replacement holding similar effects to its scheduled NP counterpart, either from scratch or from its counterpart as a precursor (Francis and Smith 2022). Pairing this understanding with varying national NP monitoring infrastructure and legislation, write-in and phenethylamine frequencies may reflect different national availabilities. For example, whereas 2C-B has been scheduled since 2001 and has since become an established substance such as MDMA, 2C-B-FLY is still unscheduled in the USA (de Boer and Bosman 2004).

We gathered extensive use parameters for individual NPs. Oral intake was the most favoured route of administration, reflecting their frequent sale in oral formulations, such as powders and tablets (Schmidt et al. 2011). Exemptions to the rule appear with the sublingual use of lysergamide analogues and ultra-potent NBOMes, often missold on blotter papers as LSD (Bersani et al. 2014; Zawilska et al. 2020). Users also reported diverse dosing ranges and durations of drug effects. This variation is compounded by the fact users may simply be unaware of the purity and/or the quantity of the dose taken (Brunt et al. 2017). Regardless, phenethylamines show distinctive pharmacodynamics, extending beyond the psychedelic receptome. By increasing extracellular monoamine concentrations through the inhibition of norepinephrine (NET), dopamine (DAT) and serotonin (SERT) transporters, they may result in a constellation of psychostimulant-like effects outside of their hallucinogenic potential (Han and Gu 2006). Prior work has attributed these features to greater odds of physical harm (Sexton et al. 2019), findings which we reiterate in the present study, as shown by higher ratings of overall physical AEs and seizures. Legislative stances towards NPs over the years have been largely guided by high profile intoxication with NBOMEs and 2C-X derivatives with similar clinical presentations such as tachycardia, hypertension and convulsions (Dean et al. 2013; Palamar et al. 2016a, b; Hondebrink et al. 2020). However, outside of seizures, we are unable to quantify the severity of these effects for users. For example, often a distinctive feature of serotonergic psychedelics is the ‘body-load’; transient somatic symptoms (nausea, discomfort, vomiting) which often accompany the onset of their effects (Dos Santos et al. 2018).

Care should be taken prior to attributing patterns of specific psychological AEs, in light of our finding of no overall significant differences. While one can state certain deleterious traits such as observed heightened anxiety following lysergamide use, or low mood following phenethylamines are accounted by differences in subjective high quality or mood sequelae stemming from compound-dependent neurotoxicity (Zwartsen et al. 2018; Xu et al. 2019; Asanuma et al. 2020), psychedelic experiences often comprise volatile emotional states (Brouwer and Carhart-Harris 2020). Observed outcomes may therefore be defined by an amalgamation of non-pharmacological contextual factors (Hartogsohn 2016). Despite much having been written about the relative risks of each class, it is currently unknown whether any hold particular therapeutic benefits. Novel tryptamines such as 5-MeO-DMT and purportedly high-risk phenethylamines such as DOI have been documented to be neuroplasticity-inducing the latter appearing among surveyed microdosers (Hutten et al. 2019). As such, prospective studies set prior to use may be able to further clearer indices of their relative harms and benefits while issues pertaining to ecology and the legal status of NPs in such studies may be circumvented by employing volunteer-orientated citizen-science designs (Silvertown 2009) such as those used in microdosing studies (Szigeti et al. 2021).

Functional differences in signalling cascades stemming from structural differences are hypothesised to relate to the unique nature of narrative drug experiences (Zamberlan et al. 2018). Using pairwise comparisons and a ML approach, we demonstrated that despite users holding similar motivations to use 2C-B, 4-AcO-DMT and 1P-LSD, the phenomenological markers of these were not correspondent. Entheogenic features such as *oceanic boundlessness* and *dread of ego dissolution* were rated significantly less for 2C-B than for 4-AcO-DMT and 1P-LSD. Characterised as an entactogen with psychedelic-like effects, observational studies have demonstrated 2C-B only produces mild psychedelic effects. As with other entactogens such as 2C-E, 4-FA and MDMA, its effects are limited to perceptual alterations and pseudohallucinations (Papaseit et al. 2018; Kuypers et al. 2019; Papaseit et al. 2020; Studerus et al. 2021). These descriptions may be exemplified by the absence of dose-dependent effects, endorsement of *euphoria* as a motivation by 49% of users and its reiterated use at music events (Palamar et al. 2016a, b). Consequently, that what distinguishes certain phenethylamines from tryptamines and lysergamides may not be a question of experience quality, but rather depth. In this study, our XGBoost approach served as a useful proof of concept for the distinction of drug effects using ML. Our model yielded better class prediction for 4-AcO-DMT and 1P-LSD, with the former showing the highest specificity and sensitivity. Looking to their pharmacology, each is seemingly prodrugs of their respective progenitor’s psilocybin and LSD, and is reported to produce comparable effects to their predecessors (Coney et al. 2017; Palamar and Acosta 2020). 4-AcO-DMT (*O*-Acetylpsilocin) is an acetylated equivalent of psilocybin’s primary bioactive metabolite psilocin (Madsen et al. 2019) previously suggested to be a suitable substitute for psilocybin for clinical use (Nichols 1999), with 1P-LSD (1-Propionyl-d-lysergic acid diethylamide) similarly hydrolysed to LSD upon intake (Brandt et al. 2016; Grumann et al. 2020). These findings may extend to other closely related homologues such as 4-HO-MET or ALD-52, albeit with variable potency. Users choosing these substances may therefore be guided by their accessibility and familiarity of effects. Pointing to this, 20% of 4-AcO-DMT users and 19.7% of 1P-LSD in our study reported using them as legal substitutes for classical compounds.

The results reported herein should be considered in the light of some key limitations. Our self-selected homogenous sample of NP users may not be representative of the general population. Whereas we collected contextual demographic variables for reference and controlled for age and sex in our regression analyses, larger cross-sectional studies specifically aimed at collecting unexamined sociodemographic variables such as ethnicity and income may prove to demonstrate their influence on AE outcomes. Furthermore, NP prevalence may vary according to the study timeframe, user location and degree of prior experience. As such, subsets of experienced use may have clouded our capacity to detect class-specific associations and/or inflated dosages. While precautions were taken during XGboost development to diminish model variability, several caveats should be considered. Training was performed on a small subset of users, in the absence of external validation with an external independent sample for each compound, nor was the predictive viability of the 5D-ASC cross-examined. Adding to this, resampling methods such as SMOTE do not take into consideration that neighbouring examples can be from other classes, which may further diminish the occurrence of useful edge cases. While our chosen trio hold close structural and phenomenological resemblance to other members of their NP class, our examination is a first steppingstone towards more comprehensive evaluations. Future work will assess model specificity across a greater range of NP representatives, including exemptions to the rule such as DiPT, reported to primarily produce auditory distortions (Carbonaro et al. 2013).

In conclusion, the present work provides a dictionary of use characteristics for structurally independent novel psychedelics and demonstrates NPs may be discerned by their entheogenic properties. Future legislative approaches should take into consideration the overlapping nature of novel homologues with classical predecessors of clinical use. Work should continue to establish reference points to salient NP subclasses, as to confirm the veracity of these findings. Follow-up studies should aim to employ a dual-prong fishing approach in the form of online surveys harmonising free narratives alongside validated retrospective assessments for a particular compound.

## Supplementary Information

Below is the link to the electronic supplementary material.

Supplementary file1 (DOCX 1.11 MB)
