# Supplementary material for: The use patterns of novel psychedelics: experiential fingerprints of substituted phenethylamines, tryptamines and lysergamides
Source: Psychopharmacology (Berl). 2022 Apr 30;239(6):1783–96. doi: 10.1007/s00213-022-06142-4 (PMC9166850; doi:10.1007/s00213-022-06142-4)
Supplement: Supplementary file 1 — Supplementary file1 (DOCX 1.11 MB) [file 213_2022_6142_MOESM1_ESM.docx]

# Supplementary Materials

**Subjective effect scale descriptions.**

The 5D-ASC dimension “Oceanic Boundlessness” (27 items) comprises feelings of derealisation and depersonalization associated with positive emotional experiences. Its lower order scales consist of “experience of unity,” “spiritual experience,” “blissful state,” and “insightfulness.” The dimension “Anxious Ego Dissolution” (21 items) feeling of ego-loss and self-control phenomena associated with anxiety. Its lower-order scales are “disembodiment,” “impaired control of cognition,” and “anxiety.” “Visionary Restructuralisation” (18 items) describes perceptual changes, consisting of the subscales “complex imagery,” “elementary imagery,” “audio-visual synesthesia,” and “changed meaning of percepts.” Lastly, “Auditory Alterations” and “Reduction of Vigilance” consist of 15 items and 12 items respectively.

The 48-item Addiction Research Centre Inventory (ARCI) was employed (Martin, Sloan et al. 1971) was employed as to further delineate potential differences between psychedelic NPS families and other recreational drug classes. A 49-item true-false scale, each scale of ARCI is defined according to Amphetamine-like effects are defined by feelings of increased energy, sense of wellbeing), Benzedrine-like effects reflect increased energy, intellectual productivity, Morphine-Benzedrine-like effects comprise pleasant somatic experiences such as euphoria, Lysergic Acid Diethylamide-like effects are associated to dysphoria, somatic discomfort, and Pentobarbital-Chlorpromazine-Alcohol-like effects are described as sedation, psychomotor retardation. Due to the retrospective nature of the survey, several adaptations were made to suit the study design, as per prior work (Bieber, Fernandez et al. 2008). We removed two items explicitly worded in the present tense (“*Answering these questions was* *very easy today*” and “*It seems I'm spending longer than I should on each of these questions*”) and modified “*I would be happy all the time if I felt as I do now”* to reflect a recalled experienced. A true-false item checking the retrospective nature of the responses following completion of the ARCI was used as a fail-safe to exclude incorrect responses.

**Motives and setting scale descriptions.**

Respondents were asked to rank the importance of the listed motives for their personal use from 1 to 3 (1 for the main motivation, 3 for the 3rd main motivation). The scale was designed as an extension of the 18-item reasons for drug-use scale by Boys et al. (Boys, Marsden et al. 2001). Additional items comprised motives related to social context, self-exploration, and escapism, stemming from qualitative interviews (Prepeliczay 2016), in addition to the option for respondents to write in an unlisted motive. The importance of each motive ascribed to a particular substance was determined by generating a total rank order score for each scale item. Further, a binary yes/no option was provided to state whether they had reached their main motivation.

Users were asked to list in which environment their most recent full-dose experience took place. Listed options consisted of a party setting, festival setting, home setting, work setting, ceremonial or spiritual setting, or another setting.

**Supplementary frequentist analyses.**

We investigated drug-dependent (2C-B, 4-AcO-DMT and 1P-LSD) differences in ascribed use motive importance and subjective effect scores (5D-ASC, ARCI) by employing a set of Kruskal–Wallis one-way analysis of variance tests. Where applicable, post-hoc multiple comparisons were performed using Bonferroni-corrected (*p* < 0.017*)* pairwise Dunn’s tests. Detailed results for subjective effects can be found in Table S5 alongside mean scores and *p* value outcomes of one-tailed t-tests *t* (176,102,59) against zero to allow for comparison with previous datasets.

**XGboost model**

***Data pre-processing***

To minimise the influence of outliers on predictive performance, we employed RobustScaler (Scikit-learn) to normalise our features. By scaling each feature according to its median and IQR, we preserved the relative relationship of these datapoints to other values. In addition to being small, our training dataset was subject to unbalanced target classes. This can result in frequency biasing of modelling accuracy, thus leading to high probability of misclassification of the minority class as compared to the majority class. For our training data, we therefore chose to resample our minority classes using SMOTE (Synthetic Minority Oversampling TEchnique), which consists of synthesizing elements for the minority class by computing the k-nearest neighbours (in feature space) of randomly selected minority points (Chawla, Bowyer et al. 2002) while matching the majority class by random undersampling.

***Model description***

Within a dataset holding n samples and *m* features, *K* additive functions are used by XGboost to generate output predictions, using the estimation below:

${y^{^}}_{j}=\sum_{k=1}^{K} f_{k}(x_{i}),$

Whereby  $f_{k}\in\{f(x)=\omega_{q}\}(q:R^{m}\to T,\omega\in R^{T})$ is the regression tree’s space, q denotes the independent structure of each tree with *T* leaves. Each *f_k_* corresponds to a single tree’s q and leaf weights *ω*. To learn the set of functions, the following regularized objective is minimized.

$L=\sum_{i} l({y^{^}}_{i},y_{i})+\sum_{k} \Omega(f_{k}),$

where $\Omega(f)=\gamma T+\frac{1}{2}\lambda{\|\omega\|}^{2}$, *l* denotes the model loss function, and Ω denotes the regularized term.

For tasks involving classification, true positives (TP), true negatives (TN), false positives (FP), and false negatives (FN) provide an indication of the classifier’s performance against trusted external judgments. The terms positive and negative refer to the classifier's prediction (sometimes known as the expectation), and the terms true and false refer to whether that prediction corresponds to the external (actual) observation. For each of the following metrics we also provided *macro-averages* to define overall model performance. Macro averaging reduces multiclass predictions down to pairwise sets of binary predictions, calculating the corresponding metric for each of the binary cases, and then averaging the results together.

***Accuracy***

The overall model accuracy was defined as such:

$\frac{(TP+TN)}{(TP+FP+FN+T\{P)}$

***Precision.***

Precision (or specificity) is the ratio of correctly predicted positive observations to the total predicted positive observations and can be listed as such:

$\frac{TP}{TP +FP}$

The metric can be exemplified by the question: how many of the *returned* drug labels corresponded to a true instance?

***Recall.***

Recall (or sensitivity) provides the ratio of correctly predicted positive observations to the all observations in the actual class :

$\frac{TP}{TP +FN}$

This is contextualised by the question: how many true instances of drug were successfully found (recalled)?

***F1-score*.**

The F-measure or balanced F-score (F1 score) is the harmonic mean of precision and recall:
$\frac{TP}{TP+\frac{1}{2}(FP+FN)}$

The F1 score conveys the balance between the precision and the recall.

***Feature importance.***

Mean feature importance, i.e. gain (increase in accuracy brought by a feature to the branches it occurred on) was determined by their occurrence frequency across all training partitions. The measures is based on the number of times a variable is selected for booster tree splitting, weighted by the squared improvement to the model because of each split, and averaged over all trees.

**Supplementary findings: motives** **and setting for a recent experience**

A recurrent set of motives was identified for each of the 3 drugs. For 2C-B (60.6 %), 4-AcO-DMT (79.4 %) and 1P-LSD (71.6%), users most frequently listed the option “*Help you to broaden your consciousness/take a different perspective on the world*” as motivation for their use. When prompted, most users for 2C-B (82.1%), 4-AcO-DMT (91.4%) and 1P-LSD (92.6%) stated they had achieved their main motivation. One-way Kruskal-Wallis H tests did not identify significant differences in the importance ascribed to each motive (p >0.1). The relative importance of each motive is contextualised within each drug’s motivational pattern in **Figure S3a.** Whereas a set of motives recurred across each compound, the proportion of users endorsing them varied for each.

For 2C-B, the most endorsed motives were “*Help you feel euphoric/elated*“ (43.9%), “*Induce/enhance a spiritual experience”* (28.7%), “*Enhance an activity such as listening to music or playing a game or sport”* (25%) and “*Help you enjoy the company of your friends”* (18%).

For 4-AcO-DMT, the most frequently listed motives were: ”Induce *or enhance a spiritual experience*” and *“Help you feel elated or euphoric “*(35%), “*Enhance an activity such as listening to music or playing a game or sport”* (25%)*, “Help you feel more connected to nature”* and “*Because it was a legal alternative to another drug”* (20%)*.* In these instances, 4-AcO-DMT was most frequently used as an alternative for psilocybin by users (90%).

These themes were repeated for 1P-LSD. “*Enhance an activity such as listening to music or playing a game or sport”* (33.3%)*,” Induce or enhance a spiritual experience*” (32.1%), *“Help you feel elated or euphoric“* (24.7%). Several users reported the motive “*Because it was a legal alternative to another drug”* (19.7%), usually stating 1P-LSD as an alternative to LSD (90.1%).

The setting in which users consumed the reported substance can be found in Figure S3b. Users were asked to state in which environment this recent experience occurred. For the majority of 2C-B (48.6%) and 1P-LSD (39.2%) users, these experiences took place at home with other people. In the case of 4-AcO-DMT, most users reported taking the drug at home, alone (35.6%). Of all three, 2C-B was the most frequently taken at festivals/parties/raves (10.2%).

**Supplementary findings: Nonparametric comparisons of 2C-B, 4-AcO-DMT and 1p-LSD subjective effect scores**

A set of one-way Kruskal–Wallis H tests were performed for each item of the 5D-ASC and ARCI questionnaires identifying a recurring pattern of greater, equivalent score between 4-AcO-DMT and 1P-LSD in comparison to 2C-B. These comparisons highlighted significant main effects for each of the 5 main dimensions (χ2(2) = 10.9 *p* =< 0.05 -  χ^2^(2) = 40.9 *p* =< 0.001). Bonferroni-corrected post-hoc pairwise Dunn’s tests revealed that 2C-B generated significantly lower scores of *oceanic* *boundlessness, anxious ego-dissolution* and *visionary* *restructuralization* in contrast to 1P-LSD and 4-AcO-DMT (*p*< 0.001), as well as for *auditory alterations* in comparison to 4-AcO-DMT (*p<*0.05). 4-AcO-DMT produced significantly greater scores for *reductions of vigilance (p<*0.05), in comparison to both 1P-LSD and 2C-B. There were no other differences between 4-AcO-DMT and 1P-LSD.

Examining the extended 11 sub-scales of the 5D-ASC revealed significant main effects for 10 of the 11 scales (χ^2^(2) = 10.1 *p* =< 0.01 -  χ^2^(2) = 45.8  *p* =< 0.001), with scores for *audio-visual synthaesia* not differing between the three compounds. As before, post-hoc tests indicated for all 11 subscales (*p*>0.05) 4-AcO-DMT and 1P-LSD did not significantly differ. 2C-B was found to have significantly lower scores in comparison to both 4-AcO-DMT and 1P-LSD in 7 of 11 scales (*p<*0.05). Ratings for *blissful state*, highest for 1P-LSD, were significantly greater than those for 2C-B (*p<*0.01). Levels of *anxiety* were largest for 4-AcO-DMT in contrast to 2C-B (*p<*0.05). In the same vein, 2C-B scores for e*lementary imagery* scores were significantly lower than those of 4-AcO-DMT (*p<*0.05). Similarly, repeating this approach for the ARCI demonstrated none of the compounds significantly differing in somatic nor dysphoric effects (*LSD group* scale*)*, with significant main effects for the remaining 4 scales (χ^2^(2) = 6.5 *p* =< 0.05 - χ^2^(2) = 30.4 *p* =< 0.001). Interestingly, follow-up tests yielded no significant differences (*p*>0.05) between the three in regards to euphoria (*morphine-benzedrine group scale).* However, both 1P-LSD and 4-AcO-DMT rated significantly higher for amphetamine-like effects (*amphetamine group* scale*)* in contrast to 2C-B (*p<*0.01). Lastly 2C-B produced significantly less sedation *(pentobarbital-chlorpromazine-alcohol group* scale) than 4-AcO-DMT (*p<*0.001) but not 1P-LSD (P>0.05) whereas it scored significantly less (*p*>0.05) than 1P-lSD for stimulant-like effects (*Benzedrine group scale*). Detailed results can be found in Table S4 alongside mean scores and *p* value outcomes of one-tailed t-tests *t*(176,102,59) against zero to allow for comparison with previous datasets.

**Table S1. Mean dosages (mg) listed according to listed routes of administration.**

|  |  | Overall | | Oral | | Inhalation | | Nasal | | Sublingual | | Eyedrops | | Injection | | Rectal | | Absorption | |
| --- | --- | --- | --- | --- | --- | --- | --- | --- | --- | --- | --- | --- | --- | --- | --- | --- | --- | --- | --- |
|  |  | M | SD | M | SD | M | SD | M | SD | M | SD | M | SD | M | SD | M | SD | M | SD |
| Phenethylamines | 25b-NBOME | 0.71 | 0.86 | 0.62 | 1.33 | . | . | 1.00 | . | 0.76 | 0.35 | . | . | . | . | . | . | . | . |
|  | 25c-NBOME | 0.93 | 1.33 | 0.74 | 1.22 | . | . | 0.68 | 0.46 | 0.98 | 1.42 | . | . | . | . | 4.00 | . | . | . |
|  | 25i-NBOMe | 0.87 | 0.74 | 0.78 | 0.78 | 2.00 | . | 0.90 | 0.38 | 0.91 | 0.74 | . | . | . | . | . | . | 1.00 | . |
|  | 2C-B | 26.26 | 25.61 | 25.80 | 25.63 | 23.25 | 5.38 | 28.16 | 21.96 | 20.67 | 4.04 | . | . | . | . | 41.44 | 45.74 | . | . |
|  | 2C-C | 31.60 | 16.91 | 32.15 | 17.47 | . | . | 23.33 | 2.89 | . | . | . | . | . | . | 30.00 | . | . | . |
|  | 2C-D | 32.29 | 19.35 | 32.90 | 20.12 | . | . | 20.00 | 14.14 | . | . | . | . | . | . | 35.00 | 7.07 | . | . |
|  | 2C-E | 16.82 | 10.42 | 15.79 | 6.84 | 18.33 | 7.64 | 31.14 | 31.87 | 13.25 | 7.23 | . | . | 20.00 | . | 23.00 | 9.90 | . | . |
|  | 2C-I | 18.20 | 7.62 | 18.64 | 7.29 | 22.00 | 2.83 | 16.86 | 7.69 | 0.60 | 0.14 | . | . | . | . | 22.50 | 3.54 | . | . |
|  | 2C-P | 12.15 | 18.70 | 12.57 | 20.60 | 10.00 | . | 3.50 | 4.95 | . | . | . | . | . | . | 17.50 | 3.54 | . | . |
|  | 2C-T-7 | 19.24 | 8.95 | 19.21 | 9.14 | . | . | 20.00 | . | . | . | . | . | . | . | . | . | . | . |
|  | Bromo-Dragonfly | 3.48 | 6.87 | 4.17 | 8.85 | 5.00 | . | . | . | 1.00 | 0.00 | . | . | . | . | . | . | . | . |
|  | DOB | 4.10 | 6.51 | 4.15 | 6.24 | . | . | 25.00 | . | 1.68 | 0.81 | . | . | . | . | . | . | . | . |
|  | DOC | 4.10 | 6.51 | 4.15 | 6.24 | . | . | 25.00 | . | 1.68 | 0.81 | . | . | . | . | . | . | . | . |
|  | DOI | 2.44 | 1.59 | 2.63 | 1.60 | . | . | 1.00 | . | . | . | . | . | . | . | . | . | . | . |
|  | DOM | 5.09 | 3.88 | 5.57 | 4.03 | . | . | . | . | 3.71 | 3.23 | . | . | . | . | . | . | . | . |
| Tryptamines | 4-ACO-DiPT | 20.00 | 5.48 | . | . | . | . | . | . | . | . | . | . | 22.50 | 3.54 | 20.00 | . | 25.00 | . |
|  | 4-AcO-DMT | 25.10 | 11.20 | 45.33 | 39.12 | 10.00 | . | . | . | 10.00 | . | 17.91 | 12.94 | 24.48 | 11.89 | 25.41 | 11.16 | 24.95 | 7.58 |
|  | 4-ACO-MET | 28.96 | 16.41 | 26.54 | 12.19 | 40.00 | . | 50.83 | 36.66 | 20.00 | . | . | . | . | . | 31.25 | 4.79 | . | . |
|  | 4-HO-DET | 27.14 | 6.71 | 25.77 | 4.49 | . | . | 45.00 | . | . | . | . | . | . | . | . | . | . | . |
|  | 4-HO-DiPT | 24.92 | 13.76 | 24.92 | 13.76 | . | . | . | . | . | . | . | . | . | . | . | . | . | . |
|  | 4-HO-MET | 24.55 | 8.95 | 24.60 | 8.76 | 13.50 | 2.12 | 26.25 | 11.88 | 17.00 | 3.46 | . | . | . | . | 29.17 | 5.85 | . | . |
|  | 4-HO-MiPT | 23.94 | 7.83 | 23.68 | 7.83 | . | . | 25.00 | . | 27.50 | 3.54 | . | . | . | . | 32.50 | 10.61 | . | . |
|  | 5-MEO-DALT | 24.58 | 15.81 | 26.43 | 18.13 | 23.00 | 16.81 | 21.25 | 8.54 | . | . | . | . | . | . | . | . | . | . |
|  | 5-MEO-DiPT | 13.22 | 6.28 | 12.93 | 6.44 | . | . | 17.00 | . | . | . | . | . | . | . | . | . | . | . |
|  | 5-MEO-DMT | 30.46 | 37.07 | 32.20 | 40.55 | 37.53 | 41.31 | 11.00 | 7.44 | . | . | . | . | 6.00 | 3.61 | 10.00 | . | . | . |
|  | 5-MEO-MiPT | 10.20 | 5.53 | 9.98 | 5.54 | 11.14 | 3.98 | 15.00 | 8.66 | 8.33 | 2.89 | . | . | . | . | 11.50 | 7.05 | . | . |
|  | DIPT | 54.08 | 25.65 | 54.82 | 27.67 | 60.00 | . | 40.00 | . | . | . | . | . | . | . | . | . | . | . |
|  | DPT | 65.50 | 48.57 | 88.40 | 73.84 | 43.64 | 26.93 | 70.58 | 47.02 | . | . | . | . | 58.75 | 37.50 | 55.04 | 37.01 | . | . |
|  | MiPT | 57.00 | 54.50 | . | . | . | . | . | . | . | . | . | . | 50.00 | . | 20.00 | . | . | . |
| Lysergamides | 1P-LSD | 0.17 | 0.26 | 0.18 | 0.34 | . | . | . | . | 0.17 | 0.10 | . | . | . |  |  |  |  |  |
|  | ALD-52 | 0.16 | 0.08 | 0.15 | 0.07 | . | . | . | . | 0.17 | 0.08 | . | . | . |  |  |  |  |  |
|  | AL-LAD | 0.23 | 0.19 | 0.23 | 0.24 | . | . | . | . | 0.22 | 0.10 | . | . | . |  |  |  |  |  |
|  | LSZ | 0.17 | 0.09 | 0.18 | 0.08 | . | . | . | . | 0.17 | 0.11 | . | . | . |  |  |  |  |  |

Mean dosages (M) per route of administration for each reported drug are listed alongside their standard deviation (SD).

**Table S2. Median durations listed according to route of administration.**

|  |  | Oral | | Inhalation | | Nasal | | Sublingual | | Injection | | Rectal | | Absorption | |
| --- | --- | --- | --- | --- | --- | --- | --- | --- | --- | --- | --- | --- | --- | --- | --- |
|  |  | Median | QR3 - QR1 | Median | QR3 - QR1 | Median | QR3 - QR1 | Median | QR3 - QR1 | Median | QR3 - QR1 | Median | QR3 - QR1 | Median | QR3 - QR1 |
| Phenethylamines | 25b-NBOME | 9.5 | 10-6 | . | . | 5 | 5-5 | 6 | 8-5 | . | . | . | . | . | . |
|  | 25c-NBOME | 8 | 9-6 | . | . | 7 | 8-6 | 7.5 | 10-6 | . | . | 5 | 5-5 | . | . |
|  | 25i-NBOMe | 8 | 10-6 | 5 | 5-5 | 7 | 9-6 | 8 | 10-6 | . | . | . | . | 6 | 6-6 |
|  | 2C-B | 5 | 6-4 | 3 | 3-3 | 4 | 5-3 | 7 | 10-4 | . | . | 5 | 6-4 | . | . |
|  | 2C-C | 4 | 6-4 | . | . | 2 | 4-1 | . | . | . | . | 4 | 4-4 | . | . |
|  | 2C-D | 4 | 6-3 | . | . | 2 | 8-0.25 | . | . | . | . | 4.5 | 6-3 | . | . |
|  | 2C-E | 8 | 9-6 | 7 | 10-6 | 5 | 8-3 | 7 | 8-6 | 6 | 6-6 | 7 | 9-5 | . | . |
|  | 2C-I | 6 | 8-6 | 5 | 6-4 | 4 | 6.5-4 | 6.5 | 7-6 | . | . | 6 | 6-6 | . | . |
|  | 2C-P | 14 | 16-8 | 25 | 25-25 | 5.5 | 6-5 | . | . | . | . | 12.5 | 15-10 | . | . |
|  | 2C-T-7 | 6 | 8-5 | . | . | 4 | 4-4 | . | . | . | . | . | . | . | . |
|  | Bromo-Dragonfly | 16 | 23-13 | 4 | 4-4 | . | . | 24 | 25-23 | . | . | . | . | . | . |
|  | DOB | 18 | 24-12 | . | . | 12 | 12-12 | 20 | 24-14 | . | . | . | . | . | . |
|  | DOC | 18 | 24-12 | . | . | 12 | 12-12 | 20 | 24-14 | . | . | . | . | 1 | 1 |
|  | DOI | 25 | 25-12 | . | . | 25 | 25-25 | 25 | 25-25 | . | . | . | . | . | . |
|  | DOM | 12 | 18.5-11 | . | . | . | . | 16 | 20-13 | . | . | . | . | . | . |
| Tryptamines | 4-AcO-DiPT | . | . | . | . | . | . | . | . | 0.38 | 0.5-0.25 | 0.25 | 0.25-0.25 | 0.5 | 0.5-0.5 |
|  | 4-AcO-DMT | 5 | 6-4 | 0.25 | 1.13-0.25 | 6 | 6-5 | 7 | 8-5 | . | . | 5.5 | 6-4.5 | . | . |
|  | 4-AcO-MET | 5 | 6-5 | 4 | 4-4 | 4 | 5-3 | 7 | 7-7 | . | . | 5.5 | 6-4.5 | . | . |
|  | 4-HO-DET | 5 | 6-4 | . | . | . | . | . | . | . | . | 4 | 4-4 | . | . |
|  | 4-HO-DiPT | 4.5 | 6-3 | . | . | . | . | . | . | . | . | . | . | . | . |
|  | 4-HO-MET | 5 | 6-4 | 5 | 6-4 | 4 | 5-3 | 6 | 7-5 | . | . | 5 | 6-4 | . | . |
|  | 4-HO-MiPT | 5 | 6-4 | . | . | 4 | 4-4 | 6 | 7-5 | . | . | 4 | 5-3 | . | . |
|  | 5-MEO-DALT | 4 | 5-2 | 0.25 | 1-0.25 | 1.5 | 3.5-1 | . | . | . | . | . | . | . | . |
|  | 5-MEO-DiPT | 6 | 7.5-3.5 | 4 | 4-4 | 5 | 5-5 | . | . | . | . | . | . | . | . |
|  | 5-MEO-DMT | 0.5 | 5-0.25 | 0.25 | 0.5-0.25 | 1 | 1-0.5 | . | . | 1 | 1-0.5 | 4 | 4-4 | . | . |
|  | 5-MEO-MiPT | 6 | 6-5 | 5 | 6-2 | 6 | 12-4 | 6 | 6-5 | . | . | 6 | 7-5 | . | . |
|  | DPT | 6 | 8-5 | 3 | 3-3 | 24 | 24-24 | . | . | . | . | . | . | . | . |
|  | DIPT | 3 | 4-3 | 1 | 3-0.5 | 3 | 4-2 | . | . | 2 | 2.5-1.5 | 2 | 4-2 | . | . |
|  | MiPT | . | . | . | . | . | . | . | . | 0.25 | 0.25-0.25 | 0.5 | 0.5-0.5 | . | . |
| Lysergamides | 1P-LSD | 10 | 12-8 | . | . | . | . | 10 | 12-8 | . | . | . | . | . | . |
|  | ALD-52 | 9 | 11-8 | . | . | . | . | 11 | 12-10 | . | . | . | . | . | . |
|  | AL-LAD | 8 | 10-6 | . | . | . | . | 8 | 10-6 | . | . | . | . | . | . |
|  | LSZ | 8 | 8-5 | . | . | . | . | 8 | 10-6 | . | . | . | . | . | . |

Median duration of drug effects stratified according to routes of administration. IQRS (75% - 25% are provided alongside.

**Table S3. Reported routes of administration.**

|  |  | Oral | Inhalation | Nasal | Sublingual | Injection | Rectal | Absorption | Other |
| --- | --- | --- | --- | --- | --- | --- | --- | --- | --- |
| Phenethylamines | 25b-NBOMe | 44.7 | . | 2.6 | 52.6 | . | . | . | . |
|  | 25c-NBOMe | 31 | . | 13.8 | 53.4 | . | 1.7 | . | . |
|  | 25i-NBOMe | 38.7 | 0.7 | 5.1 | 53.3 | . | . | 0.7 | 1.5 |
|  | 2C-B | 85.7 | 0.9 | 10.3 | 1.4 | . | 1.7 | . | . |
|  | 2C-C | 93.2 | . | 5.1 | . | . | 1.7 | . | . |
|  | 2C-D | 86.8 | . | 7.9 | . | . | 5.3 | . | . |
|  | 2C-E | 82.7 | 2.9 | 7.9 | 4.3 | 0.7 | 1.4 | . | . |
|  | 2C-I | 82.4 | 2.2 | 11 | 2.2 | . | 2.2 | . | . |
|  | 2C-P | 83.3 | 3.3 | 6.7 | . | . | 6.7 | . | . |
|  | 2C-T-2 | 96.7 | . | 3.3 | . | . | . | . | . |
|  | Bromo-DragonFly | 76.9 | 7.7 | . | 15.4 | . | . | . | . |
|  | DOB | 68.4 | . | 2.6 | 28.9 | . | . | . | . |
|  | DOC | 79.7 | 1.4 | 1.4 | 14.9 | . | 1.4 | 1.4 | . |
|  | DOI | 81.8 | . | 9.1 | 9.1 | . | . | . | . |
|  | DOM | 71.9 | . | . | 28.1 | . | . | . | . |
| Tryptamines | 4-AcO-DiPT | 100 | . | . | . | . | . | . | . |
|  | 4-AcO-DMT | 91.6 | 1.5 | 3.7 | 1.1 | 0.4 | 1.5 | . | . |
|  | 4-AcO-MET | 83.8 | 1.4 | 8.1 | 1.4 | . | 5.4 | . | 4.5 |
|  | 4-HO-DET | 92.3 | . | 3.8 | . | . | 3.8 | . | 0.8 |
|  | 4-HO-DiPT | 100 | . | . | . | . | . | . | 10.6 |
|  | 4-HO-MET | 86.1 | 0.8 | 8 | 2.4 | . | 2.8 | . | . |
|  | 4-HO-MiPT | 94.2 | . | 1.9 | 1.9 | . | 1.9 | . | 3.8 |
|  | 5-MeO-DALT | 57.7 | 23.1 | 15.4 | . | . | . | . | . |
|  | 5-MeO-DiPT | 84.2 | 10.5 | 5.3 | . | . | . | . | . |
|  | 5-MeO-DMT | 7.4 | 69.1 | 8.5 | . | 3.2 | 1.1 | . | . |
|  | 5-MeO-MiPT | 84.9 | 5.9 | 2.5 | 2.5 | . | 3.4 | . | . |
|  | DiPT | 19.7 | 18.2 | 43.9 | . | 6.1 | 7.6 | . | . |
|  | DPT | 85.7 | 7.1 | 7.1 | . | . | . | . | 0.4 |
|  | MiPT | 80 | . | 20 | . | . | . | . | . |
| Lysergamides | 1P-LSD | 55 | . | . | 45 | . | . | . | . |
|  | ALD-52 | 58.8 | . | . | 41.2 | . | . | . | . |
|  | AL-LAD | 55.1 | . | . | 44.9 | . | . | . | . |
|  | LSZ | 64.3 | . | . | 35.7 | . | . | . | . |

Routes of administration employed by individual NPS users (%)

**Table S4. Raw proportions of final experience listings.**

|  | Frequency | % Total N (599) |
| --- | --- | --- |
| 2C-B | 176 | 29.4 |
| Other (self-specified) | 104 | 17.4 |
| 1P-LSD | 102 | 17 |
| 4-AcO-DMT | 59 | 9.8 |
| 4-HO-MET | 50 | 8.3 |
| 5-MeO-MiPT | 15 | 2.5 |
| 5-MeO-DMT | 13 | 2.2 |
| DOC | 10 | 1.7 |
| 4-HO-MiPT | 10 | 1.7 |
| AL-LAD | 10 | 1.7 |
| 25i-NBOMe | 9 | 1.5 |
| ALD-52 | 9 | 1.5 |
| 4-ACO-MET | 6 | 1 |
| DOB | 4 | 0.7 |
| 2C-E | 3 | 0.5 |
| DPT | 3 | 0.5 |
| 2C-T-2 | 2 | 0.3 |
| 25b-NBOMe | 2 | 0.3 |
| DOM | 2 | 0.3 |
| 4-HO-DiPT | 2 | 0.3 |
| 2C-C | 1 | 0.2 |
| 2C-D | 1 | 0.2 |
| 2C-I | 1 | 0.2 |
| 2C-T-7 | 1 | 0.2 |
| 25c-NBOMe | 1 | 0.2 |
| Bromo-DragonFly | 1 | 0.2 |
| MiPT | 1 | 0.2 |
| 4-HO-DET | 1 | 0.2 |

Frequencies and proportions (%) of recent experience responses per drug.

**Table S5. Subjective effects ratings, as measured by the 5D-ASC and ARCI.**

|  | 2C-B | 4-AcO-DMT | 1P-LSD |  |  |  |
| --- | --- | --- | --- | --- | --- | --- |
|  | (mean ± SD) | (mean ± SD) | (mean ± SD) | χ^2^(2) | *p* | P_zero_ |
| *5D-ASC Scale (% score)* |  |  |  |  |  |  |
| Oceanic boundlessness | 27 ± 18.9 | 42.5 ± 21.3*** | 40.4 ± 20.9*** | 40.9 | **<0.001** | **<0.001** |
| Anxious ego-dissolution | 6.3 ± 8.7 | 16.1 ± 18.7*** | 9.9 ± 10.8** | 26.6 | **<0.001** | **<0.001** |
| Visionary restructuralization | 37.6 ± 19.6 | 48.3 ± 21.7** | 47.4 ± 21.3** | 18.9 | **<0.001** | **<0.001** |
| Auditory alterations | 6.5 ± 9.9 | 12 ± 16.7 | 9.5 ± 9.5* | 10.9 | **<0.05** | **<0.001** |
| Reductions of vigilance | 10 ± 8.9 | 17.5 ± 15.1 *† † | 10.1 ± 10.6 | 11.7 | **<0.01** | **<0.001** |
| Experience of unity | 20.3 ± 23.6 | 40.8 ± 27.1*** | 37 ± 27.9*** | 43.3 | **<0.01** | **<0.001** |
| Spiritual experience | 21.8 ± 21.4 | 46.8 ± 29.9*** | 35.7 ± 24.1*** | 45.8 | **<0.001** | **<0.001** |
| Blissful state | 32.2 ± 26.7 | 45 ± 33.9 | 47.4 ± 31.4** | 16.6 | **<0.001** | **<0.001** |
| Insightfulness | 28 ± 25.9 | 55.1 ± 30.3*** | 49.3 ± 27.9*** | 55.8 | **<0.001** | **<0.001** |
| Disembodiment | 9 ± 17.4 | 19.7 ± 23.3*** | 17.2 ± 20.1*** | 27.5 | **<0.001** | **<0.001** |
| Impaired control and cognition | 7.3 ± 10.7 | 16.9 ± 18.1*** | 11.1 ± 11.3*** | 25.7 | **<0.001** | **<0.001** |
| Anxiety | 7.8 ± 11.9 | 18.4 ± 24.2* | 11.8 ± 14.9 | 11.5 | **<0.05** | **<0.001** |
| Complex imagery | 29.8 ± 27.2 | 48.4 ± 29.0*** | 46.7 ± 30.5*** | 31.7 | **<0.001** | **<0.001** |
| Elementary imagery | 58.7 ± 24.9 | 69 ± 27.3* | 62.5 ± 27.1 | 10.1 | **<0.01** | **<0.001** |
| Audio-visual synsthesia | 39.8 ± 32.0 | 42.8 ± 35.1 | 42 ± 33.3 | 0.4 | 0.83 | **<0.001** |
| Changed meaning of percepts | 31.1 ± 28.0 | 43.4 ± 28.9*** | 47.9 ± 30.2*** | 22.2 | **<0.001** | **<0.001** |
|  |  |  |  |  |  |  |
| *ARCI Scale (% score)* |  |  |  |  |  |  |
| Amphetamine group (A) | 39.4 ± 19.2 | 49.8 ± 22.3** | 53 ± 20*** | 30.4 | **<0.001** | **<0.001** |
| Benzedrine group (BG) | 42.4 ± 17.8 | 45.2 ± 19.4 | 49.7 ± 19.1* | 11.6 | **<0.05** | **<0.001** |
| Morphine-benzedrine group (MBG) | 49.3 ± 23.1 | 52.5 ± 26.6 | 57.1 ± 23.7 | 6.5 | **<0.05** | **<0.001** |
| Pentobarbital-chlorpromazine-alcohol group (PCAG) | 24.7 ± 19.2 | 36.9 ± 20.6***† † | 23.8 ± 18.6 | 18.8 | **<0.001** | **<0.001** |
| LSD group (LSD) | 41.7 ± 15.8 | 44.5 ± 15.8 | 38.9 ± 14.6 | 5.2 | 0.075 | **<0.001** |
| P_zero_; One-tailed T-tests (T_176_) for each drug against 0. *p*: reported *p* values for post-hoc Bonferroni corrected Dunn’s tests; **p* <0.05. ** *p* <0.01. *** *p* <0.001 compared with 2C-B; # *p* <0.05. ## *p* <0.01. ###*p<*0.001 compared with 4-AcO-DMT; † *p* <0.05. †† *p* <0.01. ††† *p* <0.001 compared with 1P-LSD. | | | | | | |

**Figure S1. Side effect types and duration.**


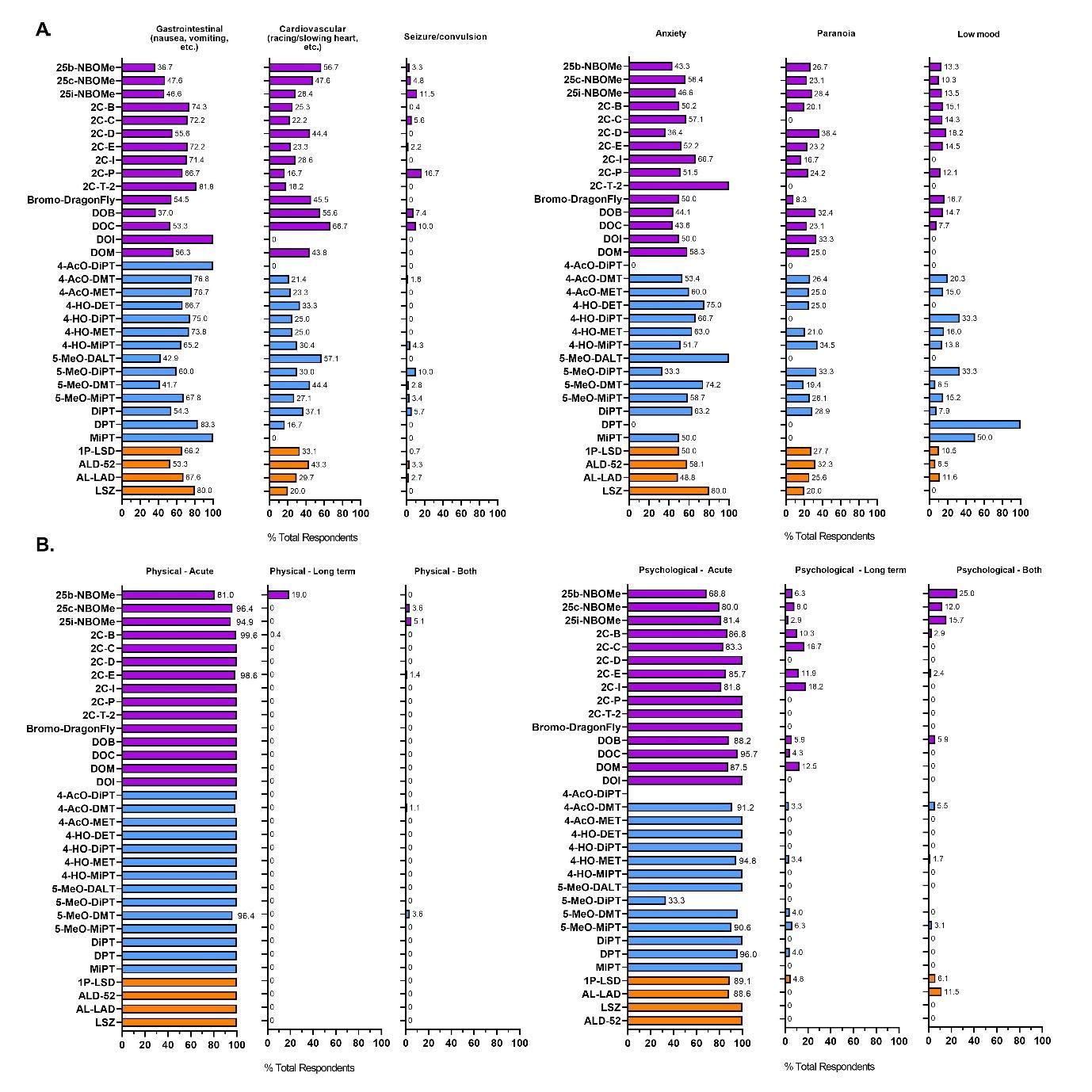


Side effects and durations are reported according to the proportions of users for each individual NPS (%).

**Figure S3. Motives and setting.**


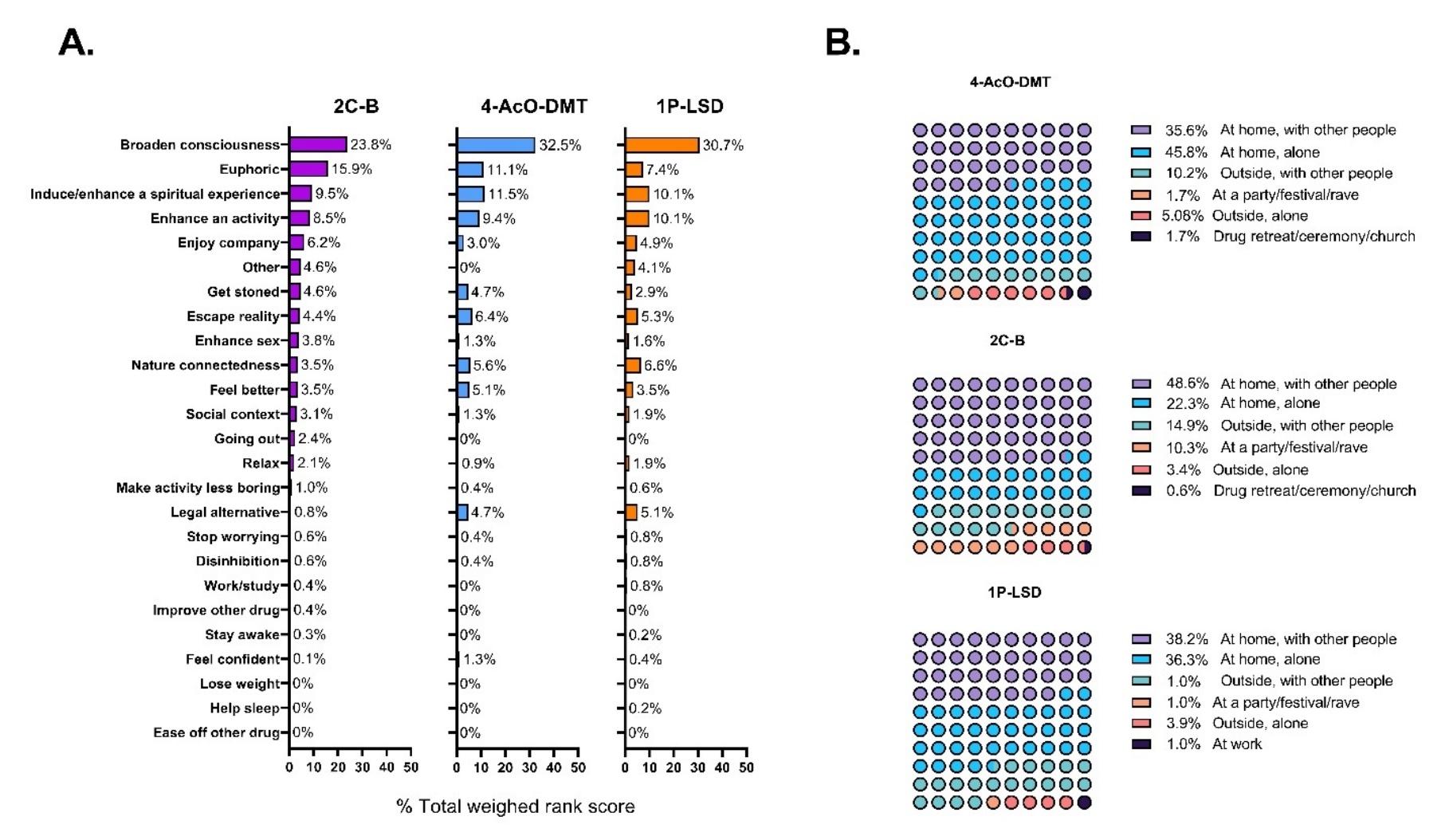


**Figure 4.** Motives and setting for the three canonical novel psychedelics 2C-B,4-AcO-DMT and 1P-LSD. **(a)** represents motives according to their percentage maximum of the total weighed rank score for **(b)** shows the percentage reported setting.

**Figure S3. Subjective effect score violin plot visualisation.**


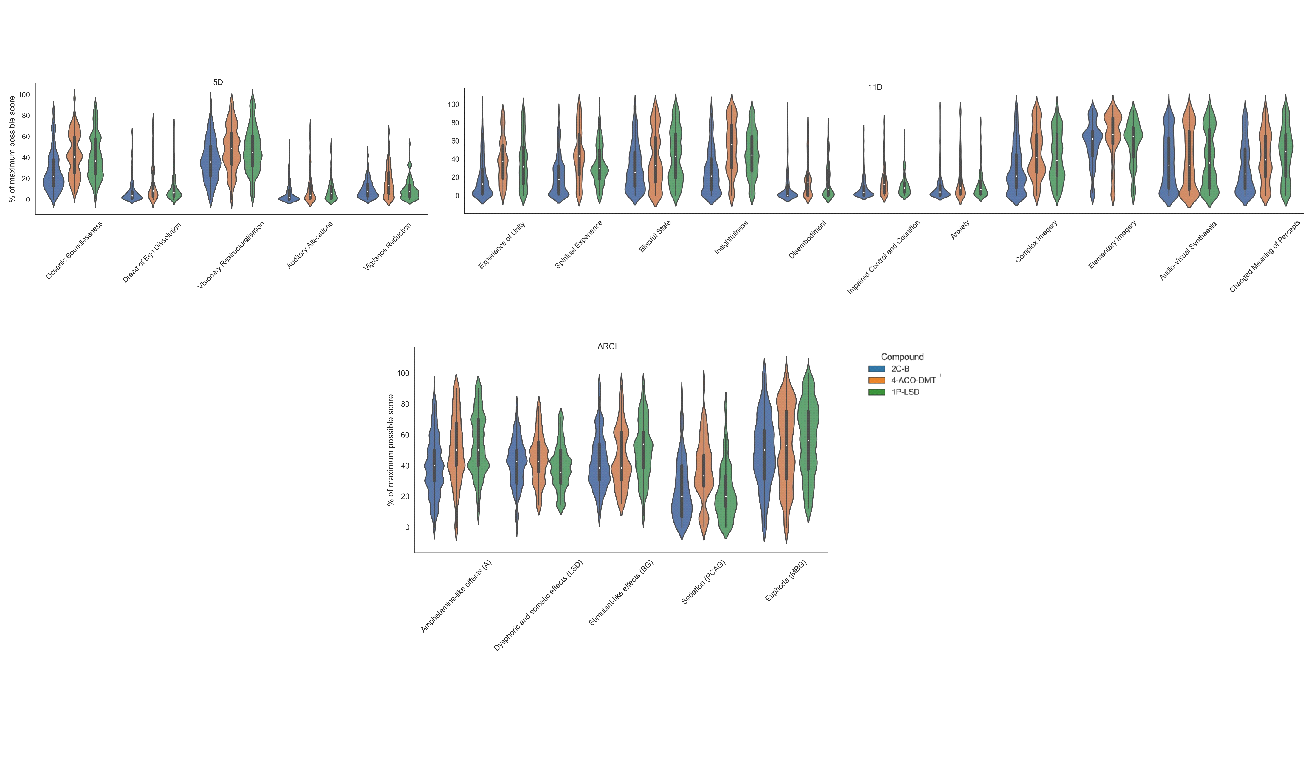


**Figure S3.** Violin plot of 5D (a) and ARCI (b) scores for 2C-B, 4-AcO-DMT and 1P-LSD. Box plots reflecting the median and interquartile range are superimposed onto kernel density estimates of each facet’s score distribution. A smoothing kernel of 0.2 was applied for this visualisation

**References**

Bieber, C. M., K. Fernandez, D. Borsook, M. J. Brennan, S. F. Butler, R. N. Jamison, E. Osgood, J. Sharpe-Potter, H. N. Thomson, R. D. Weiss and N. P. Katz (2008). "Retrospective accounts of initial subjective effects of opioids in patients treated for pain who do or do not develop opioid addiction: A pilot case-control study." Experimental and Clinical Psychopharmacology **16**(5): 429-434.

Boys, A., J. Marsden and J. Strang (2001). "Understanding reasons for drug use amongst young people: a functional perspective." Health Education Research **16**(4): 457-469.

Chawla, N. V., K. W. Bowyer, L. O. Hall and W. P. Kegelmeyer (2002). "SMOTE: synthetic minority over-sampling technique." Journal of artificial intelligence research **16**: 321-357.

Martin, W. R., J. W. Sloan, J. D. Sapira and D. R. Jasinski (1971). "Physiologic, subjective, and behavioral effects of amphetamine, methamphetamine, ephedrine, phenmetrazine, and methylphenidate in man." Clin Pharmacol Ther **12**(2): 245-258.

Prepeliczay, S. (2016). Motivationen und Morphologie des Freizeitgebrauchs von Psychedelika (LSD, Psilocybin-Pilze): eine qualitative Interviewstudie, Universität Bremen.
